# Early Childhood Education and Midlife Ideal Cardiovascular Health in a Prospective Urban Cohort

**DOI:** 10.1001/jamapediatrics.2023.4010

**Published:** 2023-10-16

**Authors:** Arthur J. Reynolds, Nishank Varshney, Suh-Ruu Ou, Rachel Kritzik, Marley Loveman-Brown

**Affiliations:** 1 Human Capital Research Collaborative, University of Minnesota, Minneapolis; 2 Humphrey School of Public Affairs, University of Minnesota, Minneapolis; 3 Now with Munroe-Myer Institute, University of Nebraska Medical Center, Omaha; 4 Institute of Child Development, University of Minnesota, Minneapolis

## Abstract

This cohort study assesses whether preschool is associated with long-term cardiovascular health as measured by the American Heart Association’s Ideal Cardiovascular Health Index.

Early childhood programs show promise in reducing cardiovascular risks and combating racial and income disparities.^1,2,3^ However, most previous studies had small sample sizes, unreplicable program elements, retrospective designs, and measurement problems.^1^ In a study of the Child-Parent Centers (CPC) program, preschool was associated with lower 30-year Framingham risk scores by age 37 years.^1^ Generalizability to broader cardiovascular health is unknown. This cohort study assessed whether preschool is associated with long-term cardiovascular health measured by the American Heart Association (AHA) Ideal Cardiovascular Health Index (iCVH)^4^ and whether educational attainment accounts for this association.

## Methods

From March 1 through June 30, 2023, we analyzed data from the Chicago Longitudinal Study, which tracks 989 children aged 3 to 4 years attending CPC preschool in 1983 to 1985 and a comparison group of 550 children who primarily attended usual early childhood education programs in randomly selected schools matched on poverty and neighborhood characteristics.^5^ Survey and health examination data were approved by the institutional review boards of Northwestern University Feinberg School of Medicine and University of Minnesota, with written and oral informed consent. We followed the STROBE guideline.

The CPC provides comprehensive educational and family support services to counteract the effects of poverty (eMethods in Supplement 1).^1,5^ After 1 to 2 years of part-day preschool, services are provided through third grade. The major long-term goal is educational attainment and greater well-being.

The iCVH is the sum of 7 positive, alterable cardiometabolic indicators and health behaviors predictive of long-term well-being (eg, healthy weight, nutrition, and blood pressure).^4^ We aligned self-report indicators against AHA’s criteria to obtain total scores from 0 to 7 (higher scores indicate greater risk) (eTable 1 in Supplement 1). Supporting validity, iCVH moderately correlated with Framingham risk score (*r* = −0.59) and in-person examination results (*r* = 0.67) and correlated as expected with self-rated health (*r* = 0.25).

We used linear regression with inverse propensity score weighting (IPW) to adjust for attrition bias and included well-established baseline covariates (eMethods in Supplement 1). Alternative estimates support robustness (eTable 2 in the Supplement 1). Analyses were conducted using SPSS, version 29. Two-tailed *P* < .05 denoted significance. The mediator was years of education.

## Results

Of 1539 participants, 1124 (73.0%) completed a survey on health and well-being (mean age, 34.9 [range, 32-37] years). Of 1042 with iCVH scores, 690 were in the program group and 352 in the comparison group. Groups were similar at baseline (Table 1). The mean (SD) iCVH score was 4.05 (0.99), with 291 participants (27.9%) having a score of 3 or lower and 309 (29.7%), a score of 5 or greater. After adjusting for baseline characteristics and IPW, CPC was associated with significantly higher iCVH score (mean [SD], 4.06 [0.96] vs 3.89 [1.03]; adjusted difference [AD], 0.17 [*P* = .01]; standardized difference [StD], 0.18) (Table 2). The pattern of CPC differences favored women (AD, 0.22 [*P* = .03]; StD, 0.23), high-poverty neighborhood status (AD, 0.23 [*P* = .03]; StD, 0.24), and high family risk status (AD, 0.19 [*P* = .02]; StD, 0.20).

**Table 1.** Characteristics of Children and Families at Follow-Up by Group

Characteristic^a^	Participants^b^		*P* value
	CPC program (n = 690)	Comparison (n = 352)	
Birth weight, mean (SD), lb	6.83 (1.26)	6.72 (1.25)	.18
Reside in neighborhood with ≥40% population at or below poverty level by age 5 y	389 (56.4)	132 (37.5)	<.001
Family risk index score by age 5 y, mean (SD)^c^	4.43 (14.03)	4.48 (14.54)	.66
Family risk index score squared	23.14 (1.64)	23.60 (1.72)	.46
Sex			
Men	305 (44.2)	178 (50.6)	.06
Women	385 (55.8)	174 (49.4)	
Race and ethnicity			
Black	642 (93.0)	332 (94.3)	.51
Hispanic and other^d^	48 (7.0)	20 (5.7)	
≥4 Family risk factors	493 (71.5)	251 (71.3)	>.99
Eligibility for subsidized meals	571 (82.8)	292 (83.0)	>.99
Single parent family status	520 (75.4)	270 (76.7)	.68
College attendance by parent	92 (13.3)	38 (10.8)	.28
Parent not employed fulltime or parttime	456 (66.1)	226 (64.2)	.58
Any child welfare case histories	22 (3.2)	15 (4.3)	.39
Chronic health condition by age 10 y	108 (15.7)	49 (13.9)	.52
Persons in original cohort with main outcome	690 (69.8)	352 (64.0)	.02
Persons in original cohort in interview at age 37 y	740 (74.8)	384 (69.8)	.03
Education by age 34 y (mediator), mean (SD), y^e^	13.00 (2.12)	12.34 (1.96)	<.001

Abbreviations: CPC, Child-Parent Centers; NA, not applicable.

^a^ Except for chronic health conditions (retrospectively reported on the midlife survey), the baseline characteristics were measured up to age 3 years or closely to time of program enrollment. The 8 family risk factors include sociodemographic factors (eg, high school dropout, not employed, and family income near the poverty level) associated with lower child well-being.

^b^ Unless otherwise indicated, data are expressed as number (percentage) of patients.

^c^ Ranges from 0 to 7, with scores of 4 or greater indicating higher risk.

^d^ Includes 1 non-Hispanic White patient.

^e^ As the hypothesized mediator, educational attainment is shown for description only.

**Table 2.** Means, Rates, and IPW-Adjusted Differences for iCVH at Age 37 Years for CPC Program and Comparison Groups in the Chicago Longitudinal Study

Sample group	iCVH score, mean					Adjusted difference (95% CI)	*P* value^a^	Standardized difference^b^	Share of group difference explained by educational attainment, %^c^
	CPC program group (n = 690)			Comparison group (n = 352)					
	Unadjusted	Adjusted		Unadjusted	Adjusted				
Total sample (n = 1042)	4.05		4.06	3.89	3.89	0.17 (0.04 to 0.31)	.01	0.18	31
Sex									
Men (n = 483)	4.01		4.06	3.91	3.91	0.15 (−0.04 to 0.34)	.14	0.12	43
Women (n = 559)	4.09		4.09	3.87	3.87	0.22 (0.02 to 0.42)	.03	0.23	22
Neighborhood poverty status^d^									
High (n = 521)	4.03		4.02	3.79	3.79	0.23 (0.02 to 0.44)	.03	0.24	27
Low (n = 521)	4.08		4.12	3.95	3.95	0.17 (−0.02 to 0.35)	.17	0.22	33
Family risk status^e^									
High (n = 744)	4.01		4.02	3.83	3.83	0.19 (0.03 to 0.36)	.02	0.20	23
Low (n = 298)	4.16		4.23	4.04	4.04	0.19 (−0.06 to 0.44)	.13	0.17	44

Abbreviations: CPC, Child-Parent Centers; iCVH, Ideal Cardiovascular Health Index; IPW, inverse propensity score weighted.

^a^ For the adjusted difference.

^b^ Standardized difference was calculated as the adjusted group difference divided by the within-group SD of iCVH for each respective group or subgroup. This SD was unadjusted and unweighted. The unadjusted SD for the total program and comparison groups were 0.96 and 1.03, respectively (nearly identical to the weighted ones). Subgroup SDs were similar to these values.

^c^ The share of the group difference explained by educational attainment is calculated as the change in the group difference from the unmediated to mediated model divided by the unmediated group difference times 100.

^d^ Measured as the percentage of population at or below the federal poverty level, with 40% or greater indicating high and less than 40% indicating low.

^e^ High family risk is defined as the presence of 4 or more risk factors vs 0 to 3 factors, based on the American Heart Association iCVH.

Years of education accounted for 31% (0.054/0.174) of the CPC-iCVH association and reduced differences to nonsignificance (Table 2). Among subgroups, percentage contribution of education was as follows: women (22%), high neighborhood poverty (27%), and high family risk status (23%).

## Discussion

Our findings support the generalizability of the association between early education and midlife cardiovascular health using a broader, positive measure. As a behavioral summary of cardiometabolic and lifestyle factors, iCVH complements other measures representing physical health. Observed differences are similar to Framingham Risk scores^1^ and family socioeconomic status.^5,6^ That education explained a sizable proportion of group differences corroborates established pathways.^6^ Limitations include group differences not being fully explained, use of self-reports, and family, school, and community factors, warranting further investigation.^2,6^ Since educational attainment is a leading social determinant of health and the most consequential outcome of early education, systemic efforts to improve educational success may promote health and well-being more generally. The pattern of benefits favored high-risk groups, suggesting that early enrichment can reduce health disparities.

## Notes

Supplement 1. **eMethods.** Definitions, Analysis, and Participants **eTable 1.** Definitions for American Heart Associations’ Ideal Cardiovascular Health (iCVH) and as Operationalized in the Chicago Longitudinal Study (CLS) **eTable 2.** Alternative Models for CPC Preschool and Ideal Cardiovascular Health at Midlife
eReferences.

Supplement 2.
Data Sharing Statement
