# Supplementary material for: Early Childhood Education and Midlife Ideal Cardiovascular Health in a Prospective Urban Cohort
Source: JAMA Pediatr. 2023 Oct 16;177(12):1350–2. doi: 10.1001/jamapediatrics.2023.4010 (PMC10580152; doi:10.1001/jamapediatrics.2023.4010)
Supplement: Supplement 1. — eMethods. Definitions, Analysis, and Participants eTable 1. Definitions for American Heart Associations’ Ideal Cardiovascular Health (iCVH) and as Operationalized in the Chicago Longitudinal Study (CLS) eTable 2. Alternative Models for CPC Preschool and Ideal Cardiovascular Health at Midlife eReferences. [file jamapediatr-e234010-s001.pdf]

Supplementary Online Content

Reynolds AJ, Varshney N, Ou S-R, Kritzik R, Loveman-Brown M. Early childhood education and midlife ideal cardiovascular health in a prospective urban cohort. *JAMA Pediatr*. Published online October 16, 2023. doi:10.1001/jamapediatrics.2023.4010

eMethods. Definitions, Analysis, and Participants

eTable 1. Definitions for American Heart Associations' Ideal Cardiovascular Health (iCVH) and as Operationalized in the Chicago Longitudinal Study (CLS)

eTable 2. Alternative Models for CPC Preschool and Ideal Cardiovascular Health at Midlife

eReferences.

This supplementary material has been provided by the authors to give readers additional information about their work.

© 2023 Reynolds AJ et al. *JAMA Pediatrics.*

eMethods. Definitions, Analysis, and Participants

AHA Definitions and Justification

The American Heart Association (AHA) defines Ideal Cardiovascular Health (iCVH) as an index of seven positive metabolic and healthy lifestyle behaviors that are associated with long-run health and well-being: Healthy weight, no smoking, normal range blood glucose (low diabetes risk), total cholesterol and blood pressure, healthy diet, and moderate or vigorous physical activity. These iCVH metrics are described by AHA as "My Life Check–Life's Simple 7."[1]

We used data from a self-reported survey on health outcomes (ages 35-37) completed by our study participants to score them on each of these metrics. Participants received a "1" on a metric if their response fell under the AHA's categorization as ideal and a "0" for intermediate or poor. However, as our data did not have the exact metrics of iCVH, close approximations were estimated (with assessment of different assumptions). Table S1 provides these operational definitions. Consistent with the original definition of iCVH, we defined each metric dichotomously as the presence or absence of ideal health, with the intermediate category scored as 0. Studies vary substantially in their definitions and scoring. Finally, we add the score across the seven metrics to compute an overall ideal index score (between 0 and 7). The mean and median value for the ideal AHA index score was four, with nearly 30% of participants having an index score of five or greater.

Prior research has found the iCVH indicators to be associated with lower levels of cardiovascular risk, particularly among Black adults.[2,3] Another meta-analysis of nine research studies shows that iCVH metrics were associated with a lower risk of cardiovascular diseases and mortality.[4] Hence, in this study, we focus our analysis on the total number of ideal indicators. One advantage of iCVH over the widely used Framingham Risk Score is that all metrics for the former are alterable for improving health and setting goals for increasing CVH in the population.[5,6]

eTable 1 provides the iCVH definitions for the criterion indicators and the measures used in this study. Prevalence rates are provided along with the iCVH means for the participants meeting each criterion. The far-right columns show that the iCVH indicators were, as expected, positively associated with self-rated health (SRH) from the midlife interview and years of education by age 34. Notably, these associations are likely underestimated given the dichotomous measurement of the cardiovascular indicators. The survey measures were corroborated with in-person health exams.[7,8] For example, the correlation between in-person examination of BMI and survey report was .85 (n = 268 participants). Supporting validity, iCVH moderately correlated with Framingham risk (r = -0.59), self-rated health (r = .25), and in-person exams at ages 37-39 (r = .67). The latter two values do not account for range restriction.

© 2023 Reynolds AJ et al. *JAMA Pediatrics.*

eTable 1. Definitions for American Heart Associations' Ideal Cardiovascular Health (iCVH) and as Operationalized in the Chicago Longitudinal Study (CLS)

Metric	AHA criterion for Ideal	CLS measure, mean age 35 yrs.	Evidence of Validity/Other	% ideal	iCVH mean	r-value SRH	r-value Edu.
**Healthy Weight**	Body Mass Index < 25.0 (Weight in kg/Height m^2)	BMI < 25.0 from interview self-report of height and weight	High correlation with exam and alternative measures	20	4.8	.12	.09
**No Smoking**	Never smoked cigarettes or quit smoking for ≥ 12 months	Never smoked based on interview self-report	Standard measure in study & correlated with most health problems	53	4.5	.07	.30
**Fasting Blood Glucose**	Less than 100 mg/dL or normal range Hemoglobin A1C; low diabetes risk	Interview self-report of not currently bothered by diabetes, not diagnosed, or being treated, allowing for write-in of other health conditions	Moderately correlated with BMI and physical inactivity; Identifies cases with less severe or no problems	95	4.1	.18	.03
**Cholesterol**	Less than 200 mg/dL (Total; untreated)	Self-report of not currently bothered, diagnosed, or being treated allowing for write-in of health conditions	May over-report health but correlated with diabetes, blood pressure, and BMI; Identifies cases with less severe/no problems; BMI proxies' total cholesterol in Framingham Risk	98	4.0	.07	.05
**Blood Pressure**	<120/<80 mm Hg, untreated Systolic/Diastolic BP	Self-report of blood pressure < 120/<80, not currently being treated or bothered by high blood pressure, allowing for write-in of other health conditions	Moderate correlation with exam; likely identifies those with less severe or no problems interfering with day-to-day health	81	4.2	.17	.08

© 2023 Reynolds AJ et al. *JAMA Pediatrics.*

**Physical Activity**	Reporting 150+ minutes of moderate, 75+ minutes of vigorous, or 150+ minutes of moderate + vigorous physical activity per week	Self-report of 150+ minutes of moderate, 75+ minutes of vigorous, or 150+ minutes of moderate + vigorous activity throughout the week over past 30 days (3 questions)	Our measure matches the AHA criterion. Greater CLS prevalence of physical activity required for jobs	32 4.5	.11 .08
**Healthy Diet Score**	Reports meeting high thresholds (daily or weekly) for 4 or 5 components of the following: fruits & vegetables, fish, whole grains/high fiber, low sodium intake, and low sugar-sweetened beverages	Reports in the interview (5 items) having 4 or 5 of the following foods available at home "always": fruits, dark green vegetables, and 1%/fat-free milk; and "never" soft drinks or fruit-flavored drinks, or fruit punch.	Meets most of the AHA components but not whole grains/high fiber. Low-fat/fat-free milk proxies healthy fats (fish). Dark green vegetables, however, is a higher standard than AHA criterion.	3.8 4.5	.11 .10

Note. iCVH sample size = 1042. Prevalence rates are based on all available data for each indicator. AHA = American Heart Association. SRH = Self-rated health from age 37 interview. Edu. = Years of education by age 34 (7 to 22; May 2014). BMI = Body Mass Index

© 2023 Reynolds AJ et al. *JAMA Pediatrics.*

Inverse Propensity Score Weighting

As a follow-up to other studies, Inverse Propensity Score Weighting (IPW) was used to adjust for potential attrition bias. Nearly 30% of the original cohort did not complete the midlife survey on health outcomes that was used to score participants on various health metrics. IPW helps remove confounding by creating a pseudo-population in which attrition from the study sample is independent of the measured confounders (baseline attributes), making the follow-up sample closely resemble that of the original sample at the beginning of the study, and thereby strengthening inferences. [9,10] The regression model included the following attrition weight variable:

Wi = 1/Pi

$$Pi\ (SR) = Constant + B_{j1}BD + B_{j2}HE + B_{j3}PR + B_{j4}SN + e$$

The predicted probabilities of sample recovery (SR; age 35-37 survey) were estimated by logistic regression (OLS regression also yields consistent estimates) with 31 input predictors hypothesized or known to be important. These included birth outcomes and demographics (BD), home environment (HE), program (PR), school, and neighborhood factors (SN). In the outcome regressions, this weight was applied such that individuals with higher weights were counted more heavily in program effect estimates (they have lower probabilities of responding to the adult survey). Those with lower weights were counted less. Analyses revealed that estimated program impacts were similar between IPW and non IPW models.

Alternative Estimates for AHA Index

Alternative models with different assumptions and definitions of ideal cardiovascular health were assessed to examine robustness of findings for the main model specification (Model 2). As shown in eTable 2, in all cases, the adjusted group differences maintained their significant associations. This included, for example, Model 4 in which BMI scores at/above 30 replaced the interview iCVH for cholesterol. This approach is consistent with the use of BMI as a stand-in for missing total cholesterol in the Framingham Risk Score calculator.[5] Alternative definitions and multiple imputation of iCVH scores also yielded similar findings.

Percent Reduction in Group Difference Associated with Educational Attainment

The share of Child-Parent Center (CPC) group differences, adjusted for baseline and IPW, explained by years of education at age 34 is shown in the Supplementary Figure. For the total sample, women and men, and residence in high poverty neighborhoods growing up (40%≥ of population below federal poverty line), education accounted for, on average, one third of the CPC-iCVH relation. Adding a second measure predictive of educational attainment—socioemotional adjustment at age 9 (6-item scale)—further increased the percent reduction estimates.

CLS Background

The Chicago Longitudinal Study (CLS) follows a same-age cohort of low-income children (N = 1539, 93% African American, 7% Latino) born in 1979-1980 who participated in federal- and state-funded early education programs beginning in preschool or kindergarten (1983-1986) in the Chicago Public School District.[11] Participants grew up in high-poverty neighborhoods in central-city Chicago and participated in one of 20 CPCs in preschool and kindergarten (N = 989) or a matched comparison group from primarily randomly selected schools offering the usual early childhood services in the district (e.g., full-day kindergarten) as well as those entering CPCs in kindergarten (N = 550). Whereas 60% of CPC participants attended full-day

© 2023 Reynolds AJ et al. *JAMA Pediatrics.*

kindergarten, all did in the comparison group. CPC participation could continue through 2nd or 3rd grade. Thus, the study assesses the benefits of the comprehensive CPC program with continuing services in the elementary grades compared to the usual early childhood services.

The key goals of the study are to: (1) assess the impacts of the CPC program on life-course development and well-being, (2) identify and better understand the pathways through which the effects of program lead to long-run health and well-being, and (3) investigate contributions of individual, family, school, and social environments from early childhood to midlife on health and well-being.[11-13]

As a prospective cohort design, participants have been followed throughout life and are currently between 43 and 44 years of age. Over the years, the CLS team has examined a wide range of predictors and outcomes that may be related to high-quality early childhood education, including parent involvement, social-emotional learning, educational attainment, employment and income, and criminal justice involvement, among others. Previous data has been collected at ages 10, 15 to 18, 18 to 24, and 26 to 28. The team has also employed various modes of data collection, including birth records, K-12 school records, parent and teacher report, participant report, and in-depth health exams for select participants.[11-13]

The current phase of the study aims to understand the association between CPC preschool participation and well-being in midlife, including physical health, economic well-being, and mental health. Nearly 1125 participants (73%) from the original sample completed a written and telephone survey on health and well-being between ages 35 and 37. Further, a sub-sample of 301 participants underwent an in-person health examination between ages 37 and 39. This study uses data on health outcomes obtained from these recent data collections.[7,8]

Two hundred and eighty-six participants (268) completed both the self-report survey by age 37 and a comprehensive in-person health exam at ages 37 to 39 (March 24, 2017 to December 21, 2019), whereby their BMI was also computed. The in-person exam occurred at the Department of Preventive Medicine Research Clinic, Northwestern University Feinberg School of Medicine in Chicago, Illinois.

We note that since our study was completed, the American Heart Association updated iCVH to include sleep quality, and is now called Life's Essential 8.[14] The need for assessing the original measure remains strong, as no studies of early childhood education have assessed it. Follow up work will investigate the new, expanded measure.

CPC Program Description

The CPC program was established in 1967 through funding from Title I of the landmark Elementary and Secondary Education Act of 1965. Initially implemented in four sites in Chicago and later expanded to 25, the program originally served families in high-poverty neighborhoods that were not reached by other early childhood programs.[15] In 2012, Arthur Reynolds at the University of Minnesota, along with participating districts, revised the CPC Program as a comprehensive school reform model to serve children in a broad variety of geographic and economic contexts.[16] Under an Investing in Innovation Grant from the U.S. Department of Education, Human Capital Research Collaborative (HCRC) began an expansion of the CPC program in 2012 in four school districts, including St. Paul, Minnesota, and Chicago, Evanston, and Normal (McLean County) in Illinois.

The CPC program aims to strengthen school achievement and overall well-being for preschool through elementary school-aged children from low-income families by way of educational enrichment, family support, health resources, and community outreach services. After 1 or 2 years of part day preschool, kindergarten through third grade services are provided. Key elements include collaborative leadership, effective learning experiences, aligned curriculum, parent involvement, professional development, and continuity and stability.

© 2023 Reynolds AJ et al. *JAMA Pediatrics.*

Continued research on the CPC preschool to 3rd grade program (P-3) and its six core elements have demonstrated effectiveness and scalability of the intervention.[16]

To promote holistic well-being, including physical health, CPCs have parent resource rooms and family programming supported collaboratively by a Parent Resource Teacher and School-Community Representative. Together, these members of the CPC leadership team conduct home visits, engage parents in the school, mobilize resources in the community, and provide referrals to health services. Parent workshops and training are a predominant element of the program, which frequently include child development, health literacy, nutrition, financial literacy, and personal development topics. Through these avenues of intervention, the CPC program is expected to promote healthy lifestyle choices and reduce cardiovascular risk over the life course.

© 2023 Reynolds AJ et al. *JAMA Pediatrics.*

eTable 2. Alternative Models for CPC Preschool and Ideal Cardiovascular Health at Midlife

**Summary statistics/model specifications**	**M0**	**M1**	**M2**	**M3**	**M4**	**M5**	**M6**	**M7**	**M8**
**Group difference, iCVH index**	**.159**	**.165**	**.174**	**.156**	**.218**	**.189**	**.192**	**.140**	**.162**
**Standard error**	**.065**	**.070**	**.070**	**.062**	**.085**	**.076**	**.094**	**.068**	**.082**
**P-value**	**.014**	**.019**	**.013**	**.011**	**.010**	**.014**	**.042**	**.041**	**.047**
**Standardized difference**	**.161**	**.167**	**.176**	**.158**	**.221**	**.191**	**.195**	**.142**	**.164**
Sample size	1042	1042	1042	1042	1042	1042	1042	1124	1124
Unadjusted iCVH mean (SD=.99; M4=1.39; M5=1.25)	4.0	4.0	4.0	4.0	3.6	3.9	3.5	4.0	3.6
Model 0. No covariates/unadjusted	Yes	No	No	No	No	No	No	No	No
Model 1. Baseline covariates	No	Yes	Yes	No	Yes	Yes	Yes	Yes	Yes
Model 2. IPW attrition adjustment (Study main model)	No	No	Yes	Yes	Yes	Yes	Yes	No	No
Model 3. IPW adjustment for program selection	No	No	No	Yes	No	No	No	No	No
Model 4. BMI≥30 changes ideal cholesterol to 0	No	No	No	No	Yes	No	Yes	No	Yes
Model 5. Cholesterol=0 if physically inactive/low diet score	No	No	No	No	No	Yes	Yes	No	No
Model 6. Model 4 + Model 5	No	No	No	No	No	No	Yes	No	No

© 2023 Reynolds AJ et al. *JAMA Pediatrics.*

Model 7. Multiple imputation with Model 1	No	No	No	No	No	No	No	Yes	Yes
Model 8. Multiple imputation with Model 4	No	No	No	No	No	No	No	No	Yes

Note. The group difference is the CPC mean minus the comparison group mean, with the standardized difference based on the within-group standard deviation (.987; unweighted and unadjusted) for the total sample. Each model assumption is displayed. Models 7 and 8 were based on multiple imputation (expectation-maximization algorithm) for the 1124 participants who responded to the survey. Input factors including baseline child and family attributes, educational attainment, and individual cardiovascular indicators. IPW = inverse propensity score weighting. iCVH = ideal cardiovascular health. (Mean age=34.9 years)

© 2023 Reynolds AJ et al. *JAMA Pediatrics.*

eReferences

1. Lloyd-Jones DM, Hong Y, Labarthe D, Mozaffarian D, Appel LJ, et al. American Heart Association Strategic Planning Task Force and Statistics Committee. Defining and Setting National Goals for Cardiovascular Health Promotion and Disease Reduction: The American Heart Association's Strategic Impact Goal through 2020 and Beyond. Circulation. 2010 Feb 2;121(4):586-613. doi: 10.1161/CIRCULATIONAHA.109.192703.

2. Fang N, Jiang M, Fan Y. Ideal Cardiovascular Health Metrics and Risk of Cardiovascular Disease or Mortality: A meta-analysis. *International Journal of Cardiology*. 2016/07/01/ 2016;214:279-283. doi:https://doi.org/10.1016/j.ijcard.2016.03.210

3. Tsao CW, Aday AW, Almarzooq ZI, et al. Heart Disease and Stroke Statistics—2022 Update: A Report from the American Heart Association. *Circulation*. 2022;145(8):e153-e639.

4. Spahillari A, Talegawkar S, Correa A, et al. Ideal Cardiovascular Health, Cardiovascular Remodeling, and Heart Failure in Blacks. *Circulation: Heart Failure*. 2017;10(2):e003682. doi:doi:10.1161/CIRCHEARTFAILURE.116.003682

5. Pencina MJ, D'Agostino RB, Larson MG, Massaro JM, Vasan RS. Predicting the 30-Year Risk of Cardiovascular Disease. *Circulation*. 2009;119(24):3078-3084. doi:doi:10.1161/CIRCULATIONAHA.108.816694. See also https://framinghamheartstudy.org/fhs-risk-functions/cardiovascular-disease-30-year-risk/

6. D'Agostino RB, Grundy S, Sullivan LM, Wilson P, for the CHD Risk Prediction Group. Validation of the Framingham Coronary Heart Disease Prediction Scores: Results of a Multiple Ethnic Groups Investigation. *JAMA.* 2001;286(2):180–187. doi:10.1001/jama.286.2.180

7. Reynolds AJ, Ou S, Eales L, Mondi CF, Giovanelli A. Assessment of a Comprehensive Early Childhood Education Program and Cardiovascular Disease Risk in Midlife. *JAMA Network Open*. 2021;4(8):e2120752-e2120752. doi:10.1001/jamanetworkopen.2021.20752

8. Reynolds AJ, Eales L, Ou S, Mondi CF, Giovanelli A. A Comprehensive, Multisystemic Early Childhood Program and Obesity at Age 37 Years. *JAMA Pediatr.* 2021;175(6):637–640. doi:10.1001/jamapediatrics.2020.6721

9. Bray B.C., Dziak J.J., Patrick M.E., Lanza S.T. Inverse Propensity Score Weighting with a Latent Class Exposure: Estimating the Causal Effect of Reported Reasons for Alcohol Use on Problem Alcohol Use 16 Years Later. Prev Sci. 2019;20(3):394-406. doi: 10.1007/s11121-018-0883-8.

10. Shiba K, Kawahara T. Using Propensity Scores for Causal Inference: Pitfalls and Tips. *Journal of Epidemiology*. 2021;31(8):457-463. doi:10.2188/jea.JE20210145

© 2023 Reynolds AJ et al. *JAMA Pediatrics.*

11. Reynolds AJ. *Success in early intervention: The Chicago Child-Parent Centers*. Lincoln: University of Nebraska Press; 2000.

12. Reynolds AJ, Ou S-R, Temple JA. A Multicomponent, Preschool to Third Grade Preventive Intervention and Educational Attainment at 35 Years of Age. *JAMA Pediatrics*. 2018:172(3): 247-256. doi:10.1001/jamapediatrics.2017.4673

13. Reynolds AJ, Ou S. Early Childhood to Young Adulthood: An Introduction to the Special Issue. *Child Youth Serv Rev.* 2010;32(8):1045-1053.

14. Lloyd-Jones DM, Allen NB, Anderson CAM, Black T, Brewer LC, et al. Life's Essential 8: Updating and Enhancing the American Heart Association's Construct of Cardiovascular Health: A Presidential Advisory from the American Heart Association. *Circulation*. 2022; 146: e18-e43. DOI: 10.1161/CIR.0000000000001078

15. Sullivan, L. M. *Let us not underestimate the children*. Glenview, IL: Scott Foresman, 1971.

16. Reynolds AJ, Hayakawa M, Candee, AJ, Englund, MM. *CPC P-3 Program Manual: Child-Parent Center Preschool-3rd Grade Program.* Minneapolis, MN: Human Capital Collaborative, University of Minnesota, 2016. http://cpcp3.org

© 2023 Reynolds AJ et al. *JAMA Pediatrics.*
