# Supplementary material for: Early Childhood Education and Midlife Ideal Cardiovascular Health in a Prospective Urban Cohort
Source: JAMA Pediatr. 2023 Oct 16;177(12):1350–2. doi: 10.1001/jamapediatrics.2023.4010 (PMC10580152; doi:10.1001/jamapediatrics.2023.4010)
Supplement: Supplement 2. — Data Sharing Statement [file jamapediatr-e234010-s002.pdf]

Data Sharing Statement

Reynolds. Early Childhood Education and Midlife Ideal Cardiovascular Health in a Prospective Urban Cohort. *JAMA Pediatr*. Published October 16, 2023.
doi:10.1001/jamapediatrics.2023.4010

Data
Data available: Yes
Data types: Data dictionary
How to access data: hcrc.umn.edu
When available: With publication

Supporting Documents
Document types: None

Additional Information
Who can access the data: approved data request
Types of analyses: approved by request
Mechanisms of data availability: submission to team
